# Construction and validity of a storyboard about breast cancer for women deprived of liberty

**DOI:** 10.1590/0034-7167-2022-0436

**Published:** 2023-11-10

**Authors:** Dayze Djanira Furtado de Galiza, Lisidna Almeida Cabral, Débora Edith Rocha Lima, Helena Alves de Carvalho Sampaio

**Affiliations:** I Universidade Estadual do Ceará. Fortaleza, Ceará, Brazil

**Keywords:** Validation Study, Breast Neoplasms, Health Education, Educational Technology, Prisoners, Estudio de Validación, Neoplasias de la Mama, Educación en Salud, Tecnología Educacional, Prisioneros, Estudos de Validação, Neoplasias da Mama, Educação em Saúde, Tecnologia Educacional, Prisioneiros

## Abstract

**Objectives:**

to build storyboards based on health literacy about breast cancer for women deprived of liberty for later production of videos and e-books.

**Methods:**

a methodological study, construction and validity of storyboards with 10 expert judges. The Educational Content Validation Instrument in Health and the Suitability Assessment of Materials were used. For validity, an agreement criterion greater than 80% was considered, verified from the Content Validation Index, with 40-100% for suitability.

**Results:**

the storyboards’ overall Content Validity Index was 0.99, combined with the measurement of suitability of 81% and readability percentage of 73.2, categorizing the material as superior and easy to read.

**Conclusions:**

the educational technology built proved to be valid and reliable, and can be transformed into an e-book and video to promote self-care for women deprived of liberty.

## RESUMO

**Objetivos:**

construir *storyboards* fundamentados no letramento em saúde sobre câncer de mama para mulheres privadas de liberdade para produção posterior de vídeos e e-books.

**Métodos:**

estudo metodológico, de construção e validação de *storyboards* com 10 juízes experts. Foram utilizados o Instrumento de Validação de Conteúdo Educativo em Saúde e o *Suitability Assessment of Materials*. Considerou-se, para validação, o critério de concordância superior a 80%, verificado a partir do Índice de Validação de Conteúdo, com 40-100% para adequação.

**Resultados:**

o Índice de Validação de Conteúdo geral dos *storyboards* foi de 0,99, aliado à mensuração da adequação de 81% e percentual de legibilidade de 73,2, categorizando o material como superior e de fácil leitura.

**Conclusões:**

a tecnologia educativa construída mostrou-se válida e confiável, podendo ser transformada em e-book e vídeo para a promoção do autocuidado de mulheres privadas de liberdade.

## RESUMEN

**Objetivos:**

construir storyboards basados en alfabetización en salud sobre cáncer de mama para mujeres privadas de libertad para la posterior producción de videos y e-books.

**Métodos:**

estudio metodológico, construcción y validación de storyboards con 10 jueces expertos. Se utilizaron el Instrumento de Validación de Contenido de Educación en Salud y el *Suitability Assessment of Materials*. Para la validación se consideró un criterio de concordancia superior al 80%, verificado a partir del Índice de Validación de Contenido, con 40-100% de adecuación.

**Resultados:**

el Índice de Validación de Contenido general de los storyboards fue de 0,99, combinado con la medida de adecuación de 81% y porcentaje de legibilidad de 73,2 categorizando el material como superior y fácil de leer.

**Conclusiones:**

la tecnología educativa construida demostró ser válida y confiable, y puede transformarse en un e-book y un video para promover el autocuidado de las mujeres privadas de libertad.

## INTRODUCTION

The world prison population of 714,000 women and girls is growing rapidly compared to the male prison population, which since 2000 has increased by more than 50%, while the male prison population has increased by around 20%^(1)^.

This condition is also found in Brazil, as the incarcerated female population increased from 5,601 to 42,355 inmates between 2000 and 2016, a growth of 525%, which represents 5.8% of the Brazilian prison population, reaching the mark of the fourth country with the most incarceration women^(2)^.

Thus, the neglect of specificities of this population’s health needs has been highlighted by the World Health Organization, making clear the interference of the prison situation in the health conditions of this population^(3)^.

This is due to the fact that the prison environment is a place prone to the existence of chronic degenerative and transmissible diseases as well as to the development or worsening of other diseases^(4, 5)^. In this regard, interventions related to encouraging self-care and health protection need to be implemented, because, although there are public policies aimed at this population, health care in the female prison system presents situations of neglect and inattention to the specific demands of women in this context, affecting with greater intensity and worsening due to the lack of access to prevention and treatment services suited to their gender specificities^(5)^.

A study related to specific themes of women, such as breast cancer, demonstrate that periodic breast examinations by the incarcerated female population do not happen, which may show the lack and/or decrease in self-care and demand for health services, even that available within the prison system. Although only 10% of women in the prison system are in the age group at greatest risk for developing breast cancer, 50 to 69 years old, what is currently recommended is the breast awareness strategy, which means being aware and alert to breast health^(6)^.

This strategy seeks to guide the female population about the usual changes in the breasts at different times of the life cycle and to recognize suspicious signs and symptoms of cancer. Thus, women should be encouraged to observe and palpate their breasts whenever they feel comfortable, without needing a specific technique or teaching a standardized method of self-examination, valuing the casual discovery of suspicious breast changes. When noticing them, women should seek medical attention as soon as possible^(7, 8, 9)^.

In this regard, educational actions that encourage self-care and recognition of suspicious signs and symptoms of breast cancer need to be implemented to prevent the discovery from being made late.

However, it is known that the incarcerated female population is composed of young, single women, from a disadvantaged social class and with low education^(10)^ and that studies show that people with these characteristics have low health literacy (HL)^(11, 12)^, making it necessary for educational interventions to be designed taking into account the level of understanding of this population.

HL corresponds to the level of understanding of health information that enables decision-making, because even individuals who know how to read and write may be unable to understand and interpret information related to health, with a mismatch between what is said and what is actually understood by users, evidencing a great knowledge gap^(13, 14)^.

Therefore, the fundamentals of HL become important tools to assist in the preparation of materials to be used during educational interventions for health promotion, mainly from people deprived of liberty, fundamentally women who need more specific health care^(15)^.

## OBJECTIVES

To build storyboards based on HL about breast cancer for women deprived of liberty for later production of videos and e-books.

## METHODS

### Ethical aspects

The research project was approved by the Research Ethics Committee of the *Universidade Estadual do Ceará*, in 2019, having complied with all the ethical precepts set forth in Resolution 466/12 of the Brazilian National Health Council.

### Study design, period, and place

This is a methodological study of construction and validity of storyboards, carried out between November 2019 and July 2020. The construction and validity of this educational technology followed the following steps: pre-production, represented by script elaboration and storyboard construction^(15)^ and validity with experts.

### Sample and inclusion, and exclusion criteria

A total of 10 judges participated in the study, selected through consultation with the Lattes curriculum, through the *Plataforma Lattes*, for internal validity, following guidelines by Pasquali^(16)^, which is from six to 20 judges. During consultation, a total of four judges who met the inclusion criteria and who had worked with video creation and/or validity were identified; therefore, priority was given to sending the invitation letter to these. As for those working with HL, there were 59. The invitation letter was sent out to all, and when a number considered accepted by the theoretical framework adopted was reached, validity was considered completed.

Thus, the following inclusion criteria were used: having a doctor’s degree and having at least one scientific production on the topic in the last 5 years. The theme considered was HL. At least one of the following situations was considered scientific production: authorship of a dissertation or thesis on the topic; dissertation or thesis guidance on the topic; authorship or co-authorship of books or book chapters on the topic; authorship or co-authorship of an article on the topic; responsibility for graduate subject (master’s or doctoral degree) on the topic.

### Study protocol

Pre-production is equivalent to the first phase of educational videos creation as these are developed from storyboards^(15)^. This time, a preliminary stage was carried out, called situational diagnosis, which took place in November 2019, with the survey, through a focus group, of health topics of interest to women deprived of liberty in a female prison in the countryside of Paraíba. From the requested themes, the most repeated ones were identified, demonstrating the majority’s interest, and selected for the elaboration of storyboards, one of which is breast cancer.

The first part of pre-production, which was script elaboration, began in January 2020, with the search and selection of national and international information on the topic, sequence definition and, finally, script writing. The technical information for its elaboration was selected on the World Cancer Research Fund International^(7)^, the International Agency for Research on Cancer^(8)^ and the National Cancer Institute^(9)^. Two scripts were prepared on the topic.

At this stage, HL fundamentals were applied to make language simple and clear, in the active voice, using short words and sentences with a maximum of 15 words, absence or clear explanation of technical terms^(17, 18)^.

After elaborating script content, an assessment was carried out using the Freeport readability index, adapted from Flesch, for Portuguese^(19)^, with the aim of ensuring that the information was at the suitable level of understanding for women deprived of liberty. This test uses a score that considers the number of syllables per word and the number of words per sentence, in order to position the analyzed texts within a 100-point scale. The test was applied to each script, using the following indexes as a reference: very easy (100-75); easy (74-50); hard (49-25); very hard (24-0)^(19)^. The syllable separator website, version 4.24, was used to calculate the necessary information^(20)^.

The second part of pre-production was the creation of story-boards. Initially, a search and selection of illustrations was carried out, also applying HL fundamentals in this selection^(17)^. The illustrations used in the storyboards, which will serve to illustrate videos or e-books, were selected in the Canva Pro program, on the Shutterstock® website, and others were produced by the authors themselves, in order to be able to illustrate exactly what would be addressed. In this way, storyboard production took place in the Canva Pro program, where selected images can be combined with the information contained in the previously prepared scripts.

HL fundamentals were also used to support the number of scenes in the storyboards, as this interferes with the duration of videos produced and also with the time required to read the e-book. The aim is that they are not long and tiring, in order to keep the audience’s attention. Furthermore, attention was paid to the amount of information that would be covered by the storyboard.

In the validity stage of the storyboards, an assessment of their content and appearance was carried out by expert judges, who were selected according to the criteria recommended by the literature^(21)^. This time, after selecting the professionals, a formal invitation was sent via email, informing about the period of participation in the study, which should take place within seven days, in addition to the link to access the invitation letter and the Informed Consent Form, which were prepared in Google Forms®. After acceptance, a new email was sent with the files of the storyboards developed, in pdf, attached.

In total, two files on the topic of breast cancer were sent. They deal with the causes of breast cancer, addressing non-modifiable and modifiable risk factors. This email also provided a link to Google Forms® with the Educational Content Validation Instrument in Health (ECVIH)^(21)^ and the Suitability Assessment of Materials (SAM), in the Portuguese version^(22)^, for carrying out script assessment regarding HL fundamentals, in addition to instructions for the interpretation of SAM topics^(23)^.

The ECVIH uses a Likert-type scale, with scores ranging from zero to two, using the following assessment options: 0 – disagree; 1- partially agree; and 2 – totally agree. In addition to this, evaluators were asked to record criticisms or suggestions for improving content^(22)^.

The SAM is an American instrument, adapted for the Portuguese language, and consists of a list or checklist with six categories, content, text understanding, graphic illustration, presentation, motivation and cultural adaptation, distributed in 22 items. A score of zero to two can be assigned to each item, with 0 being unsuitable, 1 being suitable, and 2 being totally suitable^(22)^.

### Analysis of results, and statistics

The analytical method that was used for content validity by the ECVIH was the Content Validity Index (CVI). To obtain a satisfactory CVI for each storyboard, a minimum agreement of 0.80 (domain and general) was considered, which was established by the following calculation: sum of responses “partially agree (1) and totally agree (2)” divided by the sum of all answers^(5)^.

In case of disagreement with any item, experts filled in a space for observations and suggestions for modifications. Items that obtained agreement of 0.80 or more were considered validated. Those with means between 0.75 and 0.80 were modified, according to experts’ suggestions, to be considered validated^(4, 12)^.

Regarding the analysis by SAM, the suitability of assessed material to HL fundamentals was verified. The total score was calculated from the sum of scores obtained, divided by the total number of items in the questionnaire and multiplied by 100, to transform it into a percentage. This is categorized into: 70 – 100%; superior material; 40 – 69% suitable material; and 0 – 39% unsuitable material^(10)^.

Judges’ professional profile was organized in Excel 365, to carry out descriptive analysis, with the calculation of absolute and relative frequencies, in addition to measures of central tendency (mean) and dispersion (standard deviation).

## RESULTS

**Figure 1:**
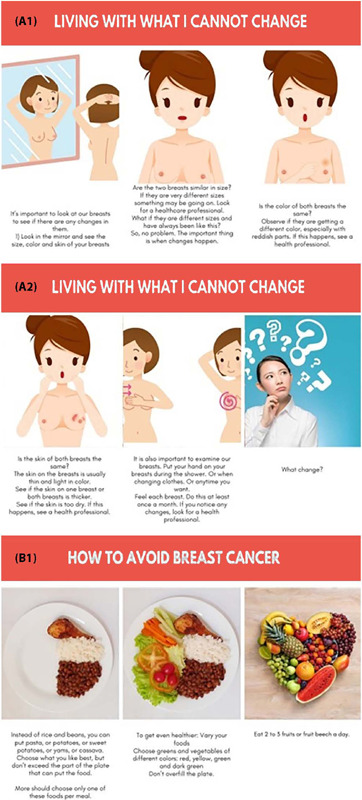
Extracted parts of storyboards

Figure 1 shows 6 scenes of each storyboard built. The first storyboard consists of 22 scenes and addresses what can be done to prevent breast cancer, focusing on non-modifiable factors such as age, skin color and genetic factor, in addition to addressing breast examination and mammography, emphasizing its importance. The second presents modifiable risk factors, such as sedentary lifestyle, obesity, poor diet and alcohol intake. In this context, two topics were addressed: activities that can be carried out within the prison environment or at home; and the importance of not gaining weight, demonstrating how to have a healthy diet from assembling a plate to the types of foods that one should avoid and those that one should eat to prevent breast cancer. This storyboard consists of 18 scenes.

A1 and A2 – screens on breast examination; B1 – strategies to avoid breast cancer.

Among the 10 participating judges, all were female. As for professional training, 07 were nurses, 02 were pharmacists, and 01 was a doctor, with training time between 10 and 38 years, with an average of 20 years (SD 9.4).

Regarding structure and presentation, the questions “interactive language, allowing active involvement in the educational process” and “suitable text size” obtained two and one “disagree” answers, respectively. However, the CVI of these two items were 0.8 and 0.9, being considered validated.

It was observed that the objective, structure/presentation and relevance domains had a CVI above 0.9, leaving the overall CVI at 0.99 (Table 1). In view of this, it can be stated that the storyboards are successfully validated by experts.

**Table 1:** Calculation of judges’ Agreement Validity Index in the objective, structure/presentation and relevance domains contained in the Educational Content Validation Instrument in Health, Fortaleza, Ceará, Brazil, 2021

Domains	Partially agree and totally agree	Content Validity Index
Objectives	100	1.00
Structure/presentation	97	0.97
Relevance	100	1.00
Total	297	0.99

Regarding the analysis performed using the SAM^(18)^, according to Table 2, two judges assessed items 5.1 and 5.3, and 4 assessed item 6.2 as “Unsuitable”. In the case of items 5.1 and 5.3, judges suggested that expressions such as “you can do it” and images with women of different age, race and sexual orientation. Thus, the text was modified, replacing the images in order to meet these requests.

**Table 2:** Judges’ assessment of content, literacy requirement, illustrations, layout and presentation, learning encouragement/motivation and cultural suitability of the two storyboards built on breast cancer and intended for women deprived of liberty, Fortaleza, Ceará, Brazil, 2021

Items assessed	Classification		
	Unsuitable %	Suitable %	Great %
1 Content			
1.1 Purpose is evident	-	10	90
1.2 Content deals with behaviors	10	30	60
1.3 Content is focused on purpose	-	40	60
1.4 Content highlights key points	-	30	70
2 Literacy requirement			
2.1 Reading level	-	60	40
2.2 Uses writing in the active voice	-	30	70
2.3 Uses vocabulary with common words in the text	-	20	80
2.4 Context comes before new information	-	30	70
2.5 Learning is facilitated by topics	-	20	80
3 Illustrations			
3.1 The illustration’s purpose referring to the text is clear	-	50	50
3.2 Types of illustrations	-	50	50
3.3 Figures/illustrations are relevant	-	40	60
3.4 Lists, tables, etc., have explanation	-	10	60
3.5 Illustrations have caption		40	50
4 Layout and presentation			
4.1 Layout feature	-	50	50
4.2 Size and font	-	40	30
4.3 Subtitles are used	-	20	50
5 Learning encouragement/motivation			
5.1 Uses interaction	20	10	70
5.2 The guidelines are specific and give examples	-	-	100
5.3 Motivation and self-efficacy	20	10	70
6 Cultural suitability			
6.1 Is similar to its logic, language and experience	-	30	70
6.2 Cultural image and examples	40	10	50

Moreover, it was requested by two evaluators that the image of the narrator of the videos be replaced by one that had characteristics more similar to women deprived of liberty, but this suggestion could not be accepted.

Regarding item 6.2, one judge suggested that the language be simple, however all the others considered the language suitable. Regarding language, analysis according to the Freeport^(18)^ readability index classified storyboard 1 as a very easy text (79.0), requiring education from 1^st^ to 5^th^ grade, storyboard 2, as an easy text (64.4), requiring schooling from 6^th^ to 9^th^ grade. The overall index, considering both storyboards, was 73.2, keeping them as an easy text.

Furthermore, the overall SAM score was found to be 81%, ranking the storyboards as superior material.

## DISCUSSION

The construction phase of storyboards is necessary for the elaboration of educational interventions aimed at producing videos or e-books, since it is in this phase that the contents, form, sequence of approach and the respective images will be decided so that the material is relevant, current and with understandable information for the target audience^(10, 13)^.

The themes of health education and technology development for the public deprived of freedom in Brazil are still very little addressed by researchers, especially for the female public. For instance^(13)^, authors discuss the literature on sexually transmitted infections within women’s prisons. Among the selected studies, only one was from Brazil and used printed material and genital organ simulators as an educational technology to address topics such as: prevention of transmission of sexual diseases/human immunodeficiency virus, safe sex practices, family planning, violence, and prevention of uterine and breast cancer. In contrast, studies developed in the United States used other types of educational technologies, such as videos and interactive computer games.

Another study that sought to identify the research carried out by Brazilian universities on female prison showed that the main themes in the area of health sciences are related to the prevalence of health problems^(14)^, demonstrating once again the scarcity of studies that address health education.

In addition to the lack of studies that address health education for women deprived of liberty, the lack of description by some studies on the validity process of technologies or educational programs used is pointed out^(13)^.

Employing validated educational technologies grants greater reliability both in using the material and in the teaching-learning process by strengthening communication in health, providing greater security for the instructions given^(15)^.

Thus, the judges showed positive responses when assessing the storyboards. The ECVIH responses elicited a CVI with high reliability and agreement within what is advocated by the literature and by studies that validate educational materials^(15)^, thus demonstrating material suitability to the intended audience.

Regarding the SAM score, the storyboards reached higher scores, therefore, suitable for use with the intended audience, showing that presentation, illustrations and layout are attractive and understandable. It is worth mentioning that validated educational materials have a higher quality in the teaching-learning process, and communication in health care reinforces the reliability of the guidelines presented and emphasizes the degree of coherence of the information in meeting the proposed objective, being an important gain for the target audience and for professional educators^(16)^.

Furthermore, judges’ recommendations contributed to material quality. The modifications contributed to improving and enriching the final product, making its applicability more concrete through the reformulation of some information and modifications of some images^(10)^.

Among judges’ recommendations, those that asked for the replacement of terms and words to make the language more similar to that of women deprived of liberty were taken into account, such as changing the expression “avoiding the disease” to “avoiding breast cancer” which is part of scene 5 of the first storyboard, as well as in the moments when what they can do, even when deprived of liberty, to avoid breast cancer, including expressions such as “you are capable” to motivate women.

Another recommendation was to replace some images with photos of women with characteristics more similar to the population for which the material is intended. The images were replaced contemplating women of different races and ages. However, it was requested by two evaluators that the image of the narrator of the videos be replaced by one that had characteristics more similar to women deprived of liberty, however the photo of the narrator in the storyboards represents the researcher of the study, who in the videos will be talking to inmates. For this reason, this suggestion could not be accepted.

Based on the assumption that knowing the reality of the public to which the educational material is intended makes the approach participatory, communicative and collective, there was a concern to include women deprived of their liberty from the beginning of the process of creating the storyboards, listening to their needs for health information and the way they would like to receive this information. Moreover, the concern to approach the themes with a language that was suitable to the level of understanding of these women allows a greater reach of this health education strategy, improving its effectiveness and reinforcing the importance of this practice, aspects also pointed out in other studies^(7, 10)^

The texts were constructed following the instructions found in two guides for digital material elaboration^(8, 17)^: use clear, short, simple and familiar words to the public; use of short sentences with a maximum of 40 to 50 characters; avoid technical terms and, if it is impossible to avoid them, explain the term; use active voice; address users when describing actions; clearly identify at least one action that users can perform, breaking it down into explicit, easy-to-follow steps; use visual aids to facilitate understanding; and follow a logical and sequential order in topic presentation, illustrating each step.

Using these fundamentals, a high level of readability was achieved, which was improved after judges validated it, resulting in an excellent level of understanding of the information contained in the storyboards. This will make it possible to understand the material both by people who have low levels of health literacy and by people who have suitable levels. Therefore, learning limitations, as a result of low schooling or low level of health literacy, were minimized, giving greater credibility to the material^(21)^.

Thus, storyboard validity demonstrated their suitability as an instrument for the production of videos that intend to guide and encourage self-care, prevention and early detection of breast cancer by women deprived of liberty through educational actions to be developed within a female prison. Similarly, the elaboration of an illustrated e-book has this functionality. Both videos and e-books can be used in different environments and realities.

However, health education actions based on health literacy are still very scarce^(23)^, especially in female prisons. A recent initiative to promote the health of incarcerated women was developed in the city of Kansas, in the United States, where health literate educational actions on cervical cancer were carried out, observing a reduction in health disparities, improvement in HL and more achievement of exams^(19)^. It was demonstrated that educational interventions designed and carried out taking into account the interest of the target population and suitable to their level of understanding can reflect in greater knowledge about the topic and better health practices.

### Study limitations

The limiting factor of this study is not having carried out validity by the target audience. This is due to the pandemic, which made access to the prison impossible by determination of the State Public Security Department, which closed the prison doors to outsiders to prevent contamination of the population deprived of liberty. It should be noted, however, that the women were heard regarding the desired contents and ways of conveying these contents before this determination so that the storyboards met their wishes. In this regard, future studies are encouraged to validate the material with the target population, in order to deepen and strengthen discussions on the topic.

### Contributions to nursing and health

In this way, we consider that the developed storyboards could be instruments to produce educational technologies, such as videos and e-books, which could encourage self-care, prevention and early detection of breast cancer by women deprived of liberty. It is expected, therefore, that the materials developed from these storyboards can encourage new habits and the implementation of self-care practices within the prison environment, enabling the early detection of cases that may occur during the period of incarceration and even after their release.

## CONCLUSIONS

Storyboards proved to be valid in terms of content and appearance, through judges’ high assessment scores, thus becoming a valid and reliable technology and enabling the creation of a tool capable of assisting in the guidance and encouragement of self-care, prevention and early detection of breast cancer by women deprived of liberty.

Video production and e-book development based on these storyboards can support health education within the prison environment, which can lead to changes in the daily lives of these women and positively influence their health.
